# Ultralow amounts of DNA from long-term archived serum samples produce quality genotypes

**DOI:** 10.1038/s41431-019-0543-x

**Published:** 2019-11-12

**Authors:** Trine B. Rounge, Marianne Lauritzen, Sten Even Erlandsen, Hilde Langseth, Oddgeir Lingaas Holmen, Randi E. Gislefoss

**Affiliations:** 1 Department of Research, Cancer Registry of Norway, Oslo, Norway; 2 Genomic Core Facility, Department of Clinical and Molecular Medicine, Norwegian University of Science and Technology, Trondheim, Norway; 3 HUNT Research Centre, Department of Public Health and General Practice, Norwegian University of Science and Technology, Levanger, Norway; 4 K.G. Jebsen Center for Genetic Epidemiology, Department of Public Health and Nursing, NTNU, Norwegian University of Science and Technology, Trondheim, Norway

**Keywords:** Epidemiology, Medical genomics, Genetics research

## Abstract

While genotyping studies are scavenging for suitable samples to analyze, large serum collections are currently left unused as they are assumed to provide insufficient amounts of DNA for array-based genotyping. Long-term stored serum is considered to be difficult to genotype since preanalytical treatments and storage effects on DNA yields are not well understood. Successful genotyping of such samples has the potential to activate large biobanks for future genome-wide association studies (GWAS). We aimed to evaluate genotyping of ultralow amounts of DNA from samples stored up to 45 years in the Janus Serum Bank with two commercially available platforms. 64 samples, with various preanalytical treatments, were genotyped on the Axiom Array from Thermo Fisher Scientific and a subset of 24 samples with slightly higher yield were genotyped on the HumanCoreExome array from Illumina. Our results showed that about 80% of the serum samples produced call rates with the Axiom arrays that would be satisfactory in GWAS. The mean DNA yield was 5.8 ng as measured with PicoGreen, 3–6% of recommended yield. The failed samples had on average lower input amounts of DNA. All serum samples genotyped on the HumanCoreExome with a standard and FFPE protocol produced GWAS satisfactory call rates, with mean 97.57% and 98.35% call rates, respectively. The mean yield was 10.65 ng, 6% of the recommendations. Successful array-based genotyping of ultralow DNA yields from serum samples stored up to 45 years is possible. These results demonstrate the potential to activate large serum biobank collections for future studies.

## Introduction

GWAS have identified associations between thousands of genetic regions and diseases [1]. The availability of high-quality DNA and cost-effective genotype readings from microarrays has been pivotal to these discoveries. Genotyping has traditionally been performed on samples with sufficient amounts of high-quality DNA, often from whole blood, producing accurate results. Many biobanks archive serum samples, a fraction of blood that does not include blood cells and clotting factors. The lack of white blood cells has deemed serum samples unsuitable for GWAS. However, serum samples may include cellular debris and low amounts of circulating DNA. Genotyping of serum DNA have been successful for single SNPs [2–5], although the DNA yields have been substantially lower than those recommended by array vendors.

To evaluate the potential of activating large collections of serum samples for array-based research, we genotyped samples from the Janus Serum Bank in Norway [6]. Specifically, we addressed whether ultralow concentrations of DNA extracted from long-term archived serum samples allowed for high-quality genotypes on the UK Biobank Axiom Array from Thermo Fisher Scientific (Waltham, US) and the HumanCoreExome array from Illumina Inc. (San Diego, US). The study was approved by Norwegian Regional Committees for Medical and Health Research Ethics (REC 2016/948).

## Material and methods

The Janus Serum Bank at the Cancer Registry of Norway contains serum samples from 318,628 Norwegian individuals collected from 1972 to 2004. The samples have been stored up to 45 years at −25 °C [6]. Preanalytical treatments, including addition of iodoacetate, clot-time, use of separating gel, and lyophilized serum, differed over the collection period.

We selected 192 samples for DNA isolation that covered all treatments and collecting periods (blood donor groups 1–6; Supplementary Table S1). DNA was isolated from 500 µL serum using the QIAamp DNA Blood Mini kit (Qiagen, Venlo, NL). DNA concentrations were measured using the Qubit™ dsDNA Assay Kit and PicoGreen™ from Thermo Fisher Scientific (Supplementary Table S1 and Supplementary Fig. S2) and 50 µL was used in the protocols.

We selected 64 samples with the largest amount of isolated DNA for genotyping on the UK Biobank Axiom Array (Axiom^®^ UKB WCSG with 845,487 probes) utilizing the GeneTitan and Axiom Whole-Genome Amplification protocol (Supplementary Fig. S1B). The protocol was modified with increased time of amplification from 23 to 48 h and hybridization from 23 to 40 h. The results from the Janus samples were compared with eight HapMap control samples with DNA inputs from 0.01 to 10 ng/μL (Supplementary Fig. S3). The Axiom protocol recommend total DNA input of 100–200 ng with a 5 ng/µL concentration. Genotypes were called according to recommendations from Thermo Fisher Scientific [7]. The Axiom best practices workflow was analyzed with 94% as the primary criteria threshold for quality control call rates (PCT-QCCR).

We selected 24 samples with the highest measured total DNA yield to be genotyped on the HumanCoreExome array from Illumina (Supplementary Fig. S1A). Samples were genotyped using two different protocols, the standard Infinium HD ultra-protocol and the Infinium HD ultra-protocol with pretreatment with the Infinium HD FFPE DNA Restore Kit from Illumina aimed to repair damaged DNA (cat no. WG-321-1002). Samples were analyzed in accordance with recommendations from the manufacturer [8]. The recommended total DNA input for the HumanCoreExome array was 200 ng, with a 50 ng/µL concentration.

## Results

The DNA yields obtained from the serum samples of all blood donor groups were substantially lower than the manufacturers arrays protocol recommendations (2.9–5.8% and 5.8% of recommended yield as measured by PicoGreen, for the Axiom and Infinium HD protocols, respectively). We measured consistently higher yield from Qubit measurement (mean 0.37 ng/µl, SD 0.25 ng/µl) compared with PicoGreen (mean 0.12 ng/µl, SD 0.12 ng/µl) (Supplementary Table S1 and Supplementary Fig. S2). The DNA were likely degraded to short fragments (Supplementary Fig. S4).

**Fig. 1:**
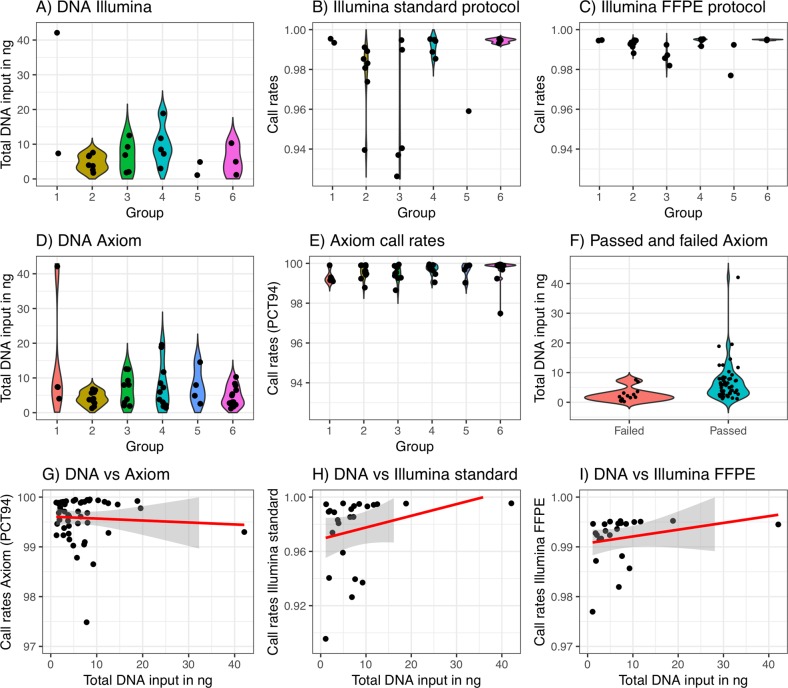
Sample characteristics and genotyping results from Janus Serum Bank samples analyzed with Axiom arrays (*n* = 64) and HumanCoreExome arrays (*n* = 24, with slightly increased yields) across six groups with different preanalytical treatment. **a,** **d** The DNA yields, obtained from 500 μL serum measured by Qubit, for the samples analyzed with Illumina and Axiom protocols, respectively. **b** Call rates obtained with standard Illumina genotyping. **c** Call rates obtained with a FFPE protocol and Illumina genotyping. **e** Call rates obtained with Axiom genotyping using 94% primary criteria threshold for quality control call rates (PCT). **f** The total DNA input in samples that failed and passed 94% PCT-QCCR. **g, h, i** The relationship between total DNA input and call rates for Axiom, standard Illumina and Illumina FFPE protocols, respectively

A total of 51 of 64 samples (79.7%) assayed with the Axiom genotyping solution passed the 94 PCT-QCCR threshold. Samples with an initial call rate lower than 82% were excluded from further analysis (*n* = 13). The Axiom average call rate was 99.5% (Fig. 1e). The average amount of analyzed DNA in the 64 samples was 5.8 ng. Mean input DNA per group is shown in Fig. 1d. The amount of input DNA was lower in failed samples compared with the samples that passed quality control (*t*-test, *P* value < 0.001; Fig. 1f). A more stringent analyses criteria, 97 PCT-QCCR (Supplementary Fig. S1C), revealed either call rates >99% or around 96%.

For the 24 sample subset with the highest DNA yield, the HumanCoreExome produced on average call rates of 97.57% and 98.35% for standard and FFPE protocol, respectively (Fig. 1b, c). The samples showed an average amount of 10.65 ng DNA. The DNA input per group is shown in Fig. 1a. Four samples (16.6%) passed quality control with the FFPE protocol that failed the regular protocol. The genotyping capture both alleles using low DNA amounts, although we do observed a slight loss of heterozygosity compared with expected allele frequencies from a high-quality DNA Norwegian reference population (Supplementary Fig. S5). We identified on average 9152 discordant markers (1.66%) for 15 sample pairs with initial call rate above 97% when comparing duplicate samples.

All protocols accurately predicted the donors’ gender and had similar performance (Supplementary Fig. S1D, E, and F**)**. There was no relationship between input DNA amounts and call rates for the samples that passed quality control (Fig. 1g–i) for any of the protocols.

## Discussion

We found that 500 μL serum archived up to 45 years yielded sufficient quality and quantity of DNA to produce quality whole-genome genotypes with both Axiom and HumanCoreExome arrays. The DNA input yields were on average 3–6% of the recommendations from the manufacturers. These findings were robust across different preanalytical treatments and preservation times as is often the case in long-term biobank archives.

Failed genotyping may be caused by a combination of low DNA quantity from serum and low DNA quality due to lengthy and varying archive periods (Supplementary Figs. S4 and S5). Therefore, a lower DNA input threshold should be assessed together with study specific DNA degradation and purity to identify individual criteria sufficient for successful genotyping. Acceptable thresholds for genotyping quality should also be considered in accordance with scientific aims, when considering DNA quantity and quality requirements. To illustrate, the observed sample failure rate may be acceptable in large multiomic settings studying common variation, and unacceptable in small studies and/or investigations of low allele frequencies. The results were not comparable across the two array types as the number of samples and selection criteria differed.

A few studies have shown that archived serum can yield sufficient DNA for analyses, with only minor signs of degradation and differences with respect to preanalytical sample handling. For example, whole-genome sequencing [9], genotyping using real-time PCR [3] and SNP array (Affymetrix) analyses [10] of Janus Serum Bank samples have been successful. However, another study concluded that genotyping with DNA extracted from serum did not provide reliable data using high-throughput multiplex approaches, but was successful using Taqman [11].

Our results may open a promising and cost-effective way to activate large serum archives for genetic studies.

## Conclusion

The successful array-based genotyping of ultralow DNA yields from archival (stored up to 45 years) serum samples shows the potential to activate large serum collections for future GWAS studies. Low DNA yield protocols did improve results somewhat, but require optimizations and additional resources. Hence, the choice of protocol and array technology should be based on available infrastructures and cost models. This study was a pilot based on a small sample size and should be repeated in a larger material.

## Supplementary information

Supplementary information
