# Supplementary material for: Ultralow amounts of DNA from long-term archived serum samples produce quality genotypes
Source: Eur J Hum Genet. 2019 Nov 12;28(4):521–4. doi: 10.1038/s41431-019-0543-x (PMC7080753; doi:10.1038/s41431-019-0543-x)
Supplement: Supplementary file 1 — Supplementary information [file 41431_2019_543_MOESM1_ESM.pdf]

Supplementary information: Ultralow amounts of DNA from long-term archived serum samples produces quality genotypes

Authors: Trine Rounge, Marianne Lauritzen, Sten Even Erlandsen, Hilde Langseth, Oddgeir Lingaas Holmen, Randi E Gislefoss

- Supplementary Table S1: Summary statistics of DNA input and genotyping results
- Supplementary Figure S1: Sample overview and comparison of call rates
- Supplementary Figure S2: Comparison of DNA input amounts measured with the Qubit and Pico Green protocols
- Supplementary Figure S3: QC gel showing fragmentation of a subset of the serum samples from the Janus Serum Bank, Norway and HapMap DNA
- Supplementary Figure S4: Fragmentation patterns of DNA from Janus Serum Bank samples
- Supplementary Figure S5: Comparison of AB allele frequencies for ~100,000 markers between Janus (N=24) and HUNT (N~55,000)
- References

Supplementary Table S1: Summary statistics of DNA input and genotyping results

Group	Collection	No of samples Affymetrix	No of samples Illumina	Storage Time in years	Sex	DNA-conc ng/µl (Qubit)	DNA-conc ng/µl (Pico Green)	Call Rate Illumina Standard	Call Rate Illumina FFPE	Call Rate Affymetrix 94PCT	Call Rate Affymetrix 95PCT	Call Rate Affymetrix 97PCT
1	1972-1978, Addition of Iodoacetate	4	2	43.38±0.36	0 F, 4 M	0.65±0.64	0.30±0.36	0.99±0.00	0.99±0.00	99.36±0.37	97.18±2.80	97.17±2.79
2	1979-1986, No additives	15	7	32.61±2.05	2 F, 13 M	0.34±0.14	0.08±0.04	0.98±0.02	0.99±0.00	99.57±0.35	98.76±2.05	96.81±2.40
3	1987-2004, Tubes with separating gel	14	5	25.68±5.12	4F, 10M	0.34±0.15	0.11±0.08	0.96±0.03	0.95±0.09	99.47±0.40	98.39±2.21	96.35±2.13
4	1973-1979, No additives or lyophilized	12	5	41.98±0.94	4F, 12 M	0.42±0.34	0.14±0.13	0.99±0.00	0.99±0.00	99.71±0.26	98.83±1.92	97.08±2.24
5	1980-1990, No additives	6	2	34.46±3.74	3F, 3M	0.25±0.14	0.11±0.10	0.93±0.04	0.98±0.01	99.61±0.40	98.46±2.59	97.55±2.65
6	1997-2004, No additives	13	3	15.85±2.44	1F, 12 M	0.34±0.16	0.09±0.06	0.99±0.00	0.99±0.00	99.63±0.70	99.37±1.41	98.82±1.87

Supplementary Figure S1: Sample overview and comparison of call rates

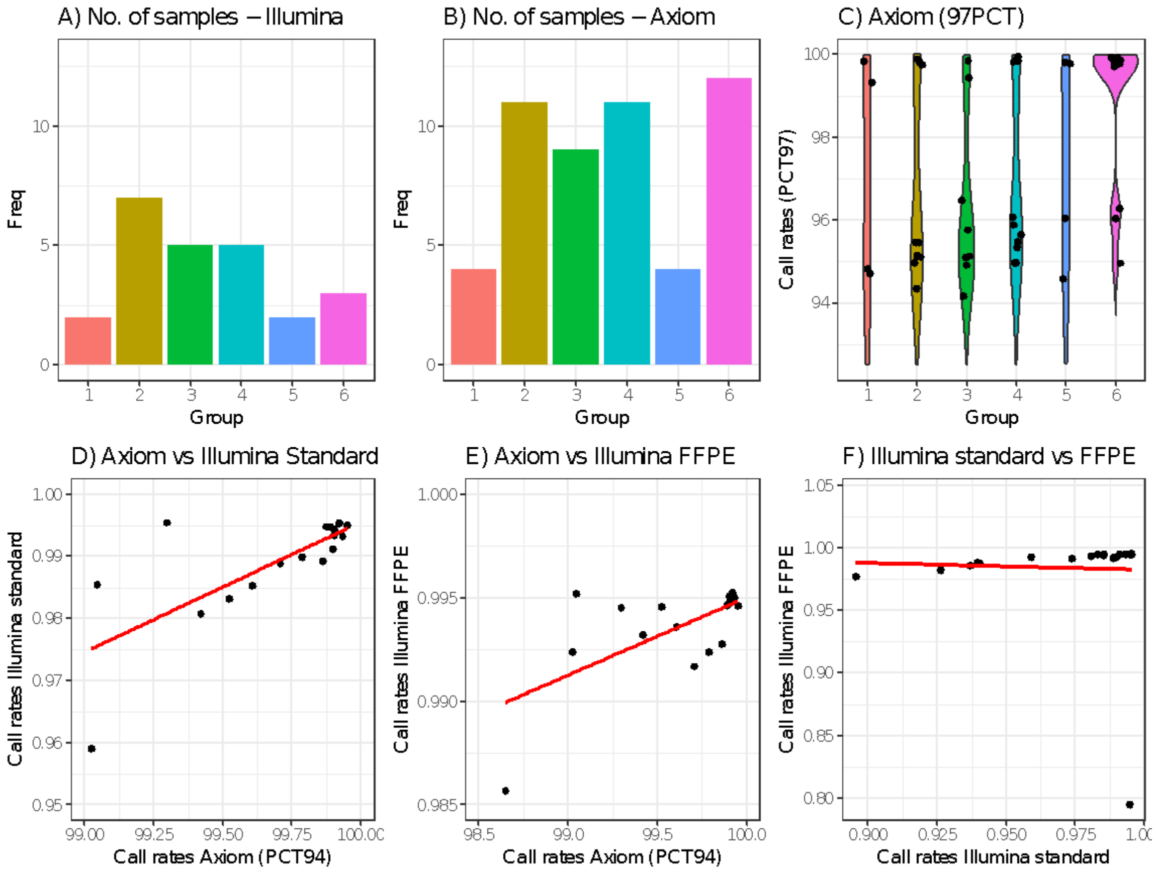

Figure S1 Legend: A) Show the number of samples analysed with Illumina arrays per blood donor group. B) Show the number of samples analysed with Axiom arrays per blood donor group. C) Distribution of call rates for the Axiom arrays using 97% primary criteria quality control call rates. Comparison of call rates for D) Axiom (94PCT) vs Illumina standard protocol, E) Axiom (94PCT) vs Illumina FFPE protocol and F) Illumina standard vs FFPE protocol.

Supplementary Figure S2: Comparison of DNA input amounts measured with the Qubit and Pico Green protocols

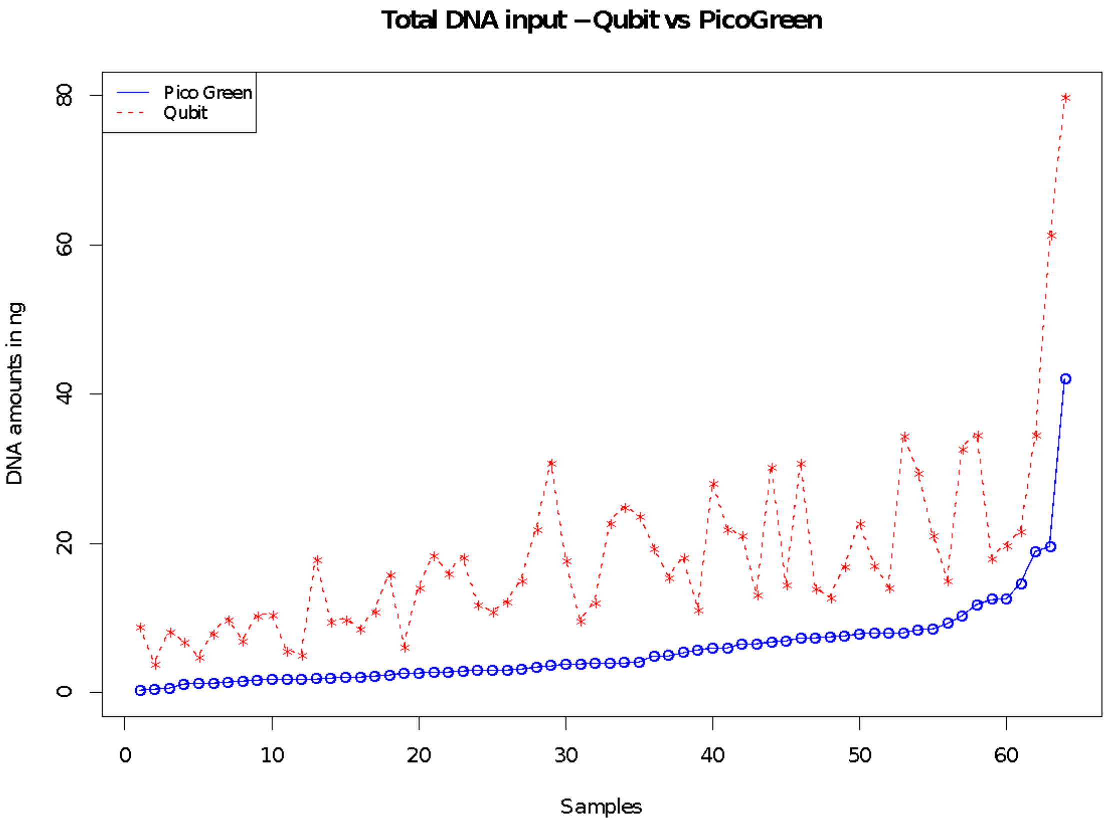

Supplementary Figure S2 Legend: Total DNA input amounts from Janus serum samples on the Axiom protocol. Samples are sorted according to Pico Green measured inputs and show that Qubit systematically overestimate DNA amounts compared to the PicoGreen protocol.

Supplementary Figure S3: QC gel showing fragmentation of a subset of the serum samples from the Janus Serum Bank, Norway and HapMap DNA

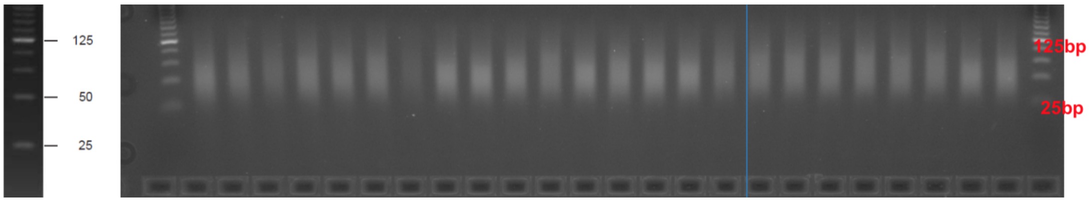

Supplementary Figure S3 Legend: Fragmentation QC gel, 4% E-gel, show comparable smears for both serum samples from the Janus Serum Bank, Norway (left of blue vertical line) and HapMap DNA (right of blue vertical line).

Supplementary Figure S4: Fragmentation patterns of DNA from Janus Serum Bank samples

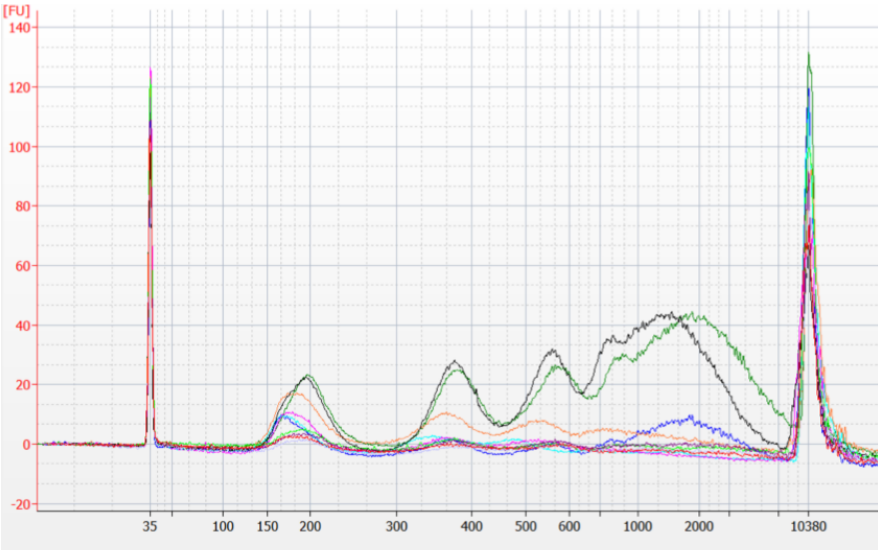

Supplementary Figure S4 Legend: Show overlay DNA traces from Bioanalyser analyses of multiple Janus Serum Bank samples. Y-axis shows the amounts of DNA in fluorescence units (FU) and X-axis show length of DNA fragments present. The up to 45-year-old samples stored at -25° C show clear signs of degradation to nucleosome size patterns. Some samples have also large amounts of longer DNA fragments.

Supplementary Figure S5: Comparison of AB allele frequencies for ~100,000 markers between Janus (N=24) and HUNT (N~55,000)

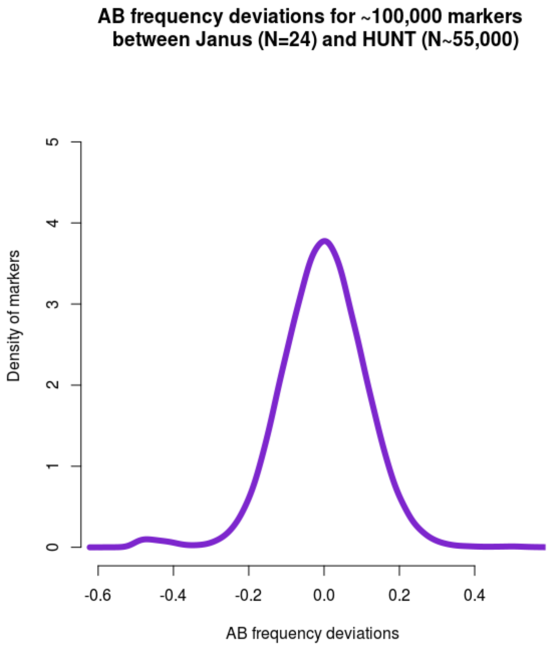

Supplementary Figure S5 Legend: The figure compares heterozygous marker frequencies (AB frequencies) between 24 individuals from the Janus cohort and ~55,000 individuals from the HUNT Study in Norway (Nielsen *et al*. 2018). The figure shows an even distribution of AB frequencies between the two studies, suggesting that the Janus genotyping do capture both alleles using low DNA amounts. The line shows frequency deviation among 134,813 heterozygous markers with a 40-60 percent frequencies. Both samples are genotyped with the same technology (HumanCoreExome). Frequencies are raw exports from Genome Studio (Illumina) pre-quality control.

References

Nielsen JB, Fritsche LG, Zhou W, et al. Genome-wide Study of Atrial Fibrillation Identifies Seven Risk Loci and Highlights Biological Pathways and Regulatory Elements Involved in Cardiac Development. Am J Hum Genet. 2018;102(1):103-115.
